# Fruits and vegetables intake and characteristics associated among adolescents from Southern Brazil

**DOI:** 10.1186/1475-2891-11-95

**Published:** 2012-11-16

**Authors:** Marta A Rieth, Marina B Moreira, Flávio D Fuchs, Leila B Moreira, Sandra C Fuchs

**Affiliations:** 1 Cardiolab-Hypertension, Hospital de Clínicas de Porto Alegre. Ramiro Barcelos 2350, Centro de Pesquisa Clínica, CEP 90035-003, Porto Alegre, RS, Brazil; 2 Postgraduate Studies Program in Cardiology, School of Medicine, Universidade Federal do Rio Grande do Sul, Ramiro Barcelos 2600, CEP 90035-003, Porto Alegre, RS, Brazil; 3 Division of Cardiology, and the National Institute for Science and Technology for Health Technology Assessment (IATS/CNPq), Hospital de Clínicas de Porto Alegre, Ramiro Barcelos 2350, Centro de Pesquisa Clínica, Cardiolab-Hipertensão, CEP 90035-003, Porto Alegre, RS, Brazil; 4 Centro de Pesquisa Clínica, 5º andar, Hospital de Clínicas de Porto Alegre, Universidade Federal do Rio Grande do Sul, Ramiro Barcellos, 2350, 90.035-003, Porto Alegre, RS, Brazil

**Keywords:** Adolescents, Fruits, Vegetables, Five-a-day, Dietary pattern, Diet

## Abstract

**Background:**

Increased body weight has been associated with an unhealthy diet, low consumption of fruits and vegetables. Our objective was to investigate whether adolescents had low intake of fruits and vegetables, and whether gender, age and education could affect the feeding patterns.

**Methods:**

A population-based sample of adolescents, aged 12–19 years, were randomly selected in southern Brazil and included in this cross-sectional study. The total daily consumption of fruits, vegetables, rice and beans were investigated in standardized household interviews, using a food frequency questionnaire and questions, being categorized as five or more servings per day as the five-a-day diet. ANOVA, ANCOVA, and modified Poisson regression were used in the analysis.

**Results:**

Adolescents (n = 568) were included, 49.5% boys, 14.3% had overweight and 8.8% obesity. Approximately 23% of participants consumed five daily servings of fruits and vegetables. It was observed that 36.7% of boys and 31.0% of girls consumed less than one serving of fruit per day, and 58.4% and 44.6%, respectively, consumed less than one serving of vegetables. The consumption of vegetables, fruits, and rice and beans were not independently associated with gender. Overweight was associated with higher intake of five-a-day, independently of confounding factors.

**Conclusions:**

Adolescents from southern Brazil have lower frequency of consumption of five servings a day of fruits and vegetables combined.

## Background

In recent decades, excessive weight gain surpassed malnutrition in adolescents worldwide [1,2]. Since the late 1970s [3], the increasing trend of overweight seems to have reached a state of equilibrium among American adolescents [4], which was similar to the observed in Brazil [5].

Several components of the diet have been investigated as determinants of the body weight gain in adolescents, including few fruits and vegetables [6], high-calorie foods [7], energetic drinks [6,8], and meals outside of the home [9]. Inadequate fruits and vegetables intake has been associated with male gender [10], increases with age [10,11], smoking [12], alcoholic beverages consumption [10], and sedentary lifestyle [13]. In addition, a direct association was detected between socioeconomic level and healthy dietary habits [10,13,14], which could influence behavioral interventions to increase fruit and vegetable intake [15,16].

The five-a-day program, initiated in the United States [17] and implemented in several countries, was created to promote fruit and vegetable consumption and augment people’s exposure in order to revert the increase of obesity-related diseases. The intake of at least five servings of fruits and vegetables a day has been recommended to reach this goal. The worldwide spread of this program led to adaptations of the main messages and some of them provide further recommendations for specific populations such as adolescents [18]. In addition, varying the colors of fruits and vegetables is more likely that adequate intake of vitamins and other nutrients is achieved [19].

However, there is a weak linkage of fruits and vegetables intake with adiposity indexes among adults [20] and it was not assessed for adolescents. We postulate that demographic and socioeconomic characteristics are some of the determinants of intake. The aim of this study was to assess the intake of fruits, vegetables and five-a-day and their association with socio-demographic characteristics among adolescents in southern Brazil.

## Material and methods

### Study design and population

A cross-sectional study was conducted on a population-based sample of adolescent boys and girls 12 to 19 years-old living in the city of Porto Alegre, southern Brazil, in 2007. The city has over 1,300,000 inhabitants and the economy is based on the third sector. Participants were selected at random through a multi-stage probability sampling based on 106 of the 2,157 census sectors (geographical subdivisions of the city, as defined by the Brazilian Institute of Geography and Statistics) [5], followed by a simple random sampling of one block in each tract, and a systematic sampling of the households from each block. All adolescents living at the eligible households were sampled. Weighting of households and effect of design were taking into account in the analysis of the data. It was used the complex sample module, of the Statistical Program for Social Sciences version 17.0 (SPSS Inc., Chicago, USA).

The sample size needed to detect a difference of one serving of fruits and vegetables a day between boys and girls in the ratio of 1:1, with 95% confidence interval and power of 80%, was estimated in 568 adolescent participants for dietary analysis. The Institution Review Board, which is accredited by the US Office of Human Research Protections, approved the protocol and participants and their legal guardians signed a consent form to participate.

### Studied variables

Subjects were interviewed at the households using standardized questionnaire regarding demographic data, lifestyle characteristics, and dietary habits. Education (years at school), smoking status (current smokers defined as ≥1 cigarettes/week for those who had smoked ≥100 cigarettes during lifetime) [21], and alcoholic beverage consumption (amount and type of alcoholic drinks consumed in the last 30 days, in order to estimate the ethanol consumed weekly [22]) were assessed. The analysis was conducted with sample stratification at the 75^th^ percentile, designating a group as having greater alcohol consumption (≥12.7 grams of ethanol/week). International Physical Activity Questionnaire (IPAQ) was used to estimate time spent in walking and all moderate-to-vigorous physical activities in the seven days prior to the interview, considering sessions that lasted over 10 minutes [23].

The interviewers were trained and a standardized questionnaire was used to collect data. Anthropometric measurements were made in duplicate, carried out with adolescents wearing light clothing and barefoot. Measurements were carried out for weight (in kilograms), measured to the nearest 100 g with a scale (Plenna® scale, model TINN 00088 Plenna - SA, São Paulo, Brazil) and height (in centimeters) to an accuracy of 0.5 cm. Body mass index (BMI) was computed as weight/height^2^ (kg/m^2^) and underweight and overweight were defined according to Cole et al. using correspondent values of BMI for children and adolescents in relation to adults cut-offs points [24]. Criteria for underweight and overweight (including obesity) for adolescents were based on the percentile curves constructed using the LMS (lambda, mu, sigma) method [25], from a curve passing through each adult cut-off point of <18.5 and ≥25.0 kg/m^2^, respectively, at age of 18 years [24,26].

### Diet evaluation

Details on the development and validation of the food frequency questionnaire (FFQ) were described elsewhere [27]. Briefly, the list generated to select food items that should be part of the FFQ were identified through 24 h dietary recall, filled by 61 adolescents selected at schools and universities in the city and in the Metropolitan area. This list was compared to that obtained in a population-based study, conducted in another city in southeastern of Brazil. The lists were similar, but 14 food items were dropped and 64 were added retaining those food items consumed by at least 5% of adolescents, in addition to food items that represent the influence of German, Italian, Japanese, and southern Brazilian cuisine. This food list was retested and additional changes were made. The FFQ inquired about the frequency and amount of consumption of 135 items, recording amount, frequency, periodicity (daily rate, weekly, monthly, or annual), and number of months in the past year each item was consumed. Each dietary item was transformed in daily ingestion (grams), and nutrients and total energy were calculated through Support to Nutrition (NUTWIN) software, developed by the Center of Computer Science in Health of Federal University of São Paulo (UNIFESP). Diets with energy intake inferior to 500 kcal or superior to 5000 kcal were considered unlikely and excluded from this analysis. In a random sample of 127 adolescents we validated the FFQ with the average of two 24 hours recalls, obtaining an attenuated correlation of 0.52 for calories [27]. The FFQ also investigated the intake of cooked items, as rice and beans, as major sources of carbohydrate and energy. The portion sizes were estimated using small, medium, and large serving spoons as a reference. Black beans are the main type of beans consumed in southern Brazil.

The questionnaire investigated cropped vegetables, defined as leaves, fruits, stems, seeds, tubers and roots used for human consumption, as a whole or in part, including: lettuce, watercress, broccoli, cauliflower, spinach, cabbage, arugula, parsley and celery, carrots, beets, zucchini, squash, cucumber, onion, and others. Fruits were identified as the pulp surrounding the plant seeds and having juice, flavor, and tastes sweet, such as: orange, bergamot, banana, apple, lemon, papaya, grapes, mango, and many others [28].

Daily intake of fruits and vegetables, in grams, was categorized on standardized servings, such as 1 medium sized fruit, ½ cup of fruits, ¼ cup of dried fruits, or 1 cup of leafy vegetables. The total of servings was computed for fruits and vegetables. The overall consumption of fruits and vegetables was categorized into five-a-day based on intake of at least five servings a day. Rice and beans, grain and fiber components were also measured by servings a day. The interviewers were trained and a standardized questionnaire was used to collect the data. Certification of the interviewers was made in loco for approximately 10% of the sampling, and additional 10% of the interviews were repeated for quality control.

### Statistical analysis

Data were entered into the Epinfo software database (Epi Info®, version 3.2.2, Atlanta, GA, USA, 2005) and checked for consistency of data entry by double entry. Crude analysis was performed using Pearson’s chi-square test (categorical variables), and analysis of variance (ANOVA) (continuous variables). Data were stratified or adjusted by gender and other confounding variables - age, education, smoking, alcoholic beverage consumption, and physical activity - in the multivariate analysis, by analysis of covariance, using the Complex samples module of the Statistical Package for Social Sciences (SPSS®, version 16, Chicago, Illinois, USA).

## Results

In the total, 568 adolescents, out of 607, aged 12 to 14 (n = 199), 15 to 17 (n = 232), and 18 to 19 years-old (n = 137), 49.5% males, and 50% white skin color were evaluated. Thirty six adolescents from 28 households declined to participate, so 94% of the eligible adolescents were enrolled. Table 1 shows that boys and girls were similar regarding age distribution, skin color, education, and smoking. However, there were different patterns of alcohol consumption, physical activity, and BMI between boys and girls. Underweight was more prevalent among girls and overweight among boys. To further explore this association, we run the analysis stratified by age, and the association between BMI and gender was statistically significant only among adolescents aged 18 to 19 years.

**Table 1:** Characteristics of adolescents from 12 to 19 years of age enrolled in SOFT study, in southern Brazil [N (%) or mean ± SD]

	**Total**	**Boys**	**Girls**	P value*
	**N = 568**	**N = 281**	**N = 287**	
Age (years)				0.8
12-14	199 (35.0)	102 (36.2)	97 (33.8)	
15-17	232 (40.8)	111 (39.5)	121 (42.2)	
18-19	137 (24.1)	68 (24.2)	69 (24.0)	
White skin color	284 (50.0)	132 (47.0)	152 (53.0)	0.15
Education (years)	7.5 ±2.5	7.5 ±2.6	7.6 ±2.5	0.8
Current smokers	57 (10.0)	23 (8.2)	34 (11.8)	0.15
Alcohol consumption in the last 30 days (g/week)^†^				0.008
No	357 (62.9)	184 (65.5)	173 (60.3)	
< 12.7	69 (12.1)	22 (7.8)	47 (16.4)	
≥ 12.7	142 (25.0)	75 (26.7)	67 (23.3)	
Physical activity				<0.001
Mild	139 (24.5)	57 (20.3)	82 (28.6)	
Moderate	269 (47.4)	115 (40.9)	154 (53.7)	
Vigorous	160 (28.2)	109 (38.8)	51 (17.8)	
Body mass index**				0.001
Underweight	45 (7.9)	11 (3.9)	34 (11.8)	
Normal	392 (69.0)	196 (69.8)	196 (68.3)	
Overweight	131 (23.1)	74 (26.3)	57 (19.9)	

* Pearson chi-squared test.

^†^ Grams of ethanol per week.

** BMI categorized as proposed by Cole et al.

The overall consumption of fruits was less than three servings a day for most of adolescents (Table 2), similar among boys and girls. The intake of at least three servings a day of vegetables was below 10%, half of the adolescents reported less than one serving a day, but it was more prevalent for boys than girls. There was no difference in the intake of five-a-day between boys and girls. The majority of boys consumed three or more servings a day of rice and beans, versus only a third of girls.

**Table 2:** Daily consumption of fruits and vegetables by adolescents enrolled in, SOFT study [N (%)]

	**Total**	**Boys**	**Girls**	P value^*^
		**(N = 281)**	**(N = 287)**	
Fruits (servings/d)				0.2
<1	192 (33.8)	103 (36.7)	89 (31.0)	
1-1.9	152 (26.8)	67 (23.8)	85 (29.6)	
2-2.9	83 (14.6)	37 (13.2)	46 (16.0)	
≥3	141 (24.8)	74 (26.3)	67 (23.3)	
Vegetables (servings/d)				0.009
<1	292 (51.4)	164 (58.4)	128 (44.6)	
1-1.9	149 (26.2)	64 (22.8)	85 (29.6)	
2-2.9	73 (12.9)	28 (10.0)	45 (15.7)	
≥3	54 (9.5)	25 (8.9)	29 (10.1)	
Five-a-day ^†^	129 (22.7)	67 (23.8)	62 (21.6)	0.5
Rice and beans (servings/d)				<0.001
<1	67 (11.8)	16 (5.7)	51 (17.8)	
1-1.9	144 (25.4)	49 (17.4)	95 (33.1)	
2-2.9	109 (19.2)	57 (20.3)	52 (18.1)	
≥3	248 (43.7)	159 (56.6)	89 (31.0)	

* Pearson chi-squared test.

† Consumption of five servings a day.

Table 3 shows that education, smoking and BMI were not associated with intake of fruits and vegetables. However, education was inversely associated whereas physical activity directly related to the consumption of rice and beans. Vigorous physical activity can probably explain the consumption of fruits per day, which could have contributed to five days. Body mass index was associated with five-a-day.

**Table 3:** **Characteristics associated with fruits and vegetables consumed per day among adolescents from Southern Brazil [mean ± SD]**^**‡**^**or n (%)**^**‡‡**^

	**Fruits**	**Vegetables**	**Five-a-day**	**Rice & beans**
Education (years)				
0-4	1.9 ±2.1	1.4 ±2.0	11 (16.9)	4.2 ±2.6
5-8	2.2 ±2.0	1.3 ±1.3	70 (23.8)	3.5 ±2.4
≥9	2.2 ±2.3	1.4 ±1.4	48 (23.0)	2.8 ±0.2
P value	0.5	0.8	0.5	<0.001
Current smoker				
No	2.2 ±2.1	1.3 ±1.4	114 (22.3)	3.3 ±2.5
Yes	2.6 ±2.7	1.6 ±1.5	15 (26.3)	3.6 ±2.2
P value	0.2	0.2	0.5	0.5
Alcohol consumption in the last 30 days (g/week)^ƒ^				
No	2.1 ±2.0	1.3 ±1.4	74 (20.7)	3.4 ±2.6
< 12.7	2.6 ±2.5	1.3 ±1.1	20 (29.0)	2.9 ±2.0
≥ 12.7	2.3 ±2.4	1.6 ±1.7	35 (24.6)	3.4 ±2.2
P value	0.09	0.06	0.3	0.4
Physical activity				
Mild	2.3 ±2.2	1.2 ±1.2	32 (23.0)	3.1 ±2.6
Moderate	1.9 ±1.9	1.4 ±1.4	51 (19.0)	3.2 ±2.3
Vigorous	2.6 ±2.5	1.5 ±1.7	46 (28.8)	3.7 ±2.6
P value	0.01	0.2	0.06	0.03
Body mass index				
Underweight	2.0 ±2.0	1.3 ±1.2	7 (15.6)	3.2 ±3.4
Normal	2.2 ±2.3	1.3 ±1.4	82 (20.9)	3.4 ±2.3
Overweight	2.4 ±2.5	1.5 ±1.5	40 (30.5)	3.2 ±2.5
P value	0.5	0.5	0.04	0.7

‡ Analysis of variance.

‡‡ Pearson chi-squared test.

† Consumption of five servings a day.

ƒ Grams of ethanol per week.

**Figure 1:**
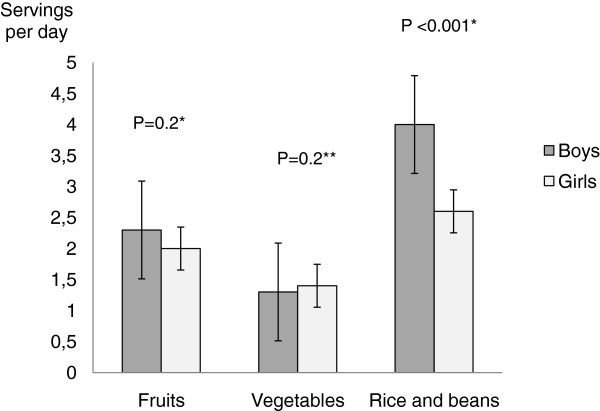
Average number of servings a day consumed by adolescents according to sex, independently of confounding factors*. * Footnote: fruits intake: adjusted for age, alcohol consumption, and physical activity; vegetables intake: adjusted for age and alcohol; rice and beans: adjusted for: and age, education, and physical activity.

Figure 1 shows that boys were more likely to consume higher number of servings a day of rice and bean than girls, independently of age, education, and physical activity. However, there was no association of gender with fruits and vegetables, even after the control for confounding factors.

**Figure 2:**
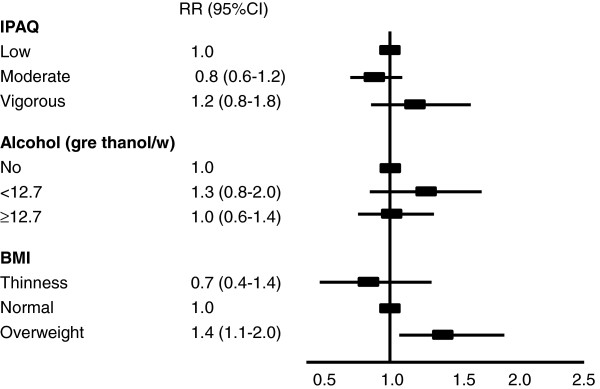
Risk ratio (95%CI) for five-a-day intake, independently of age, sex, alcohol consumption, physical activity, and BMI*. * Footnote: BMI was further categorized as normal (reference), excessive weight (overweight + obesity), and thinness.

Figure 2 demonstrates that independently of age, sex, alcohol consumption, and physical activity, adolescents who had overweight had a 1.4 prevalence of five-a-day intake in comparison to those with normal BMI.

## Discussion

This population-based survey carried out on adolescents has detected low frequency of fruits, vegetables, and five-a-day intake. Among several characteristics only overweight was associated with increased intake of five-a-day, regardless of age range, male sex, vigorous physical activity, and higher alcohol consumption. Even though, individually fruits and vegetables were not associated. Rice and beans have been consumed daily by most of the adolescents, and boys had more servings a day than girls, independently of confounding factors.

The overall five-a-day intake was lower than the reported for the American adolescents in the study EAT (Eating Among Teens), which showed that approximately a third reached the servings recommended [29]. In contrast, among middle and upper class adolescents, from private schools from Venezuela, 74% had five or more servings of fruits and 42% more than five portions of green vegetables per day [30]. On the other side, this pattern of fiber and grain consumption - typical of the Brazilian diet [31] - corroborated previous results from a population-based study in adolescents from southern Brazil [32]. However, black beans and rice were not associated with BMI, as it has been reported for adults [27].

The recommendations of the five-a-day program in Brazil have not been advertised as it was in some developed countries [33]. Information aiming the adolescent population should be provided including items belonging to the five-a-day program. White rice is an important source of energy, similar to the potato in other countries, and should be part of the dietary health plan as well as beans.

The higher prevalence of obesity among boys was different of previous studies [34,35]. Considering that the sub-analysis of BMI by gender was detected on adolescents aged 18 to 19 years, a potential explanation could be higher muscular development of boys than girls. This finding could not be discriminated by BMI that does not differentiate lean mass from fat mass.

The direct associations between socioeconomic status and consumption of fruits and vegetables or five-a-day detected in previous studies [10,13,14,35-37] were based on income or the education of a family member. In this study, instead, we evaluated the education of the adolescent. Since they have a small range of years at school, the association did not reach statistical significance. The association between age and intake of some food groups have been reported [7,38,39] and accounted for the inclusion of age as a confounding factor in this study.

The association between vigorous physical activity and higher intake of fruits seems to be part of positive habits in the adolescent’s lifestyle [40], which might be clustered with other healthy behaviors such as lower rate of smoking [41]. Even so, it was detected an association between overweight and five-a-day intake. This association could reflect that those adolescents eat more everything, including fruits and vegetables, or even that those who have the higher BMI are eating more fruit and vegetables because they are trying to lose weight. The cross-sectional design does not preclude reverse causality as a potential explanation for this finding.

This study has other limitations that deserve to be mentioned. The food intake was estimated by a FFQ over a twelve months period, and specific questions were asked regarding fruits and vegetables consumption in the 24 hours before the interview. Even both instruments being susceptible to biases, they were validated for adolescents from southern Brazil [27] in the Syndrome of Obesity and Risk Factors for Cardiovascular Disease (SOFT) study. In addition, portion size varies according to the participant and the population setting. Data from the EPIC-Norfolk study have demonstrated a discrepancy in portion sizes for different fruits and vegetables, being the servings of fruits usually greater but vegetables smaller than 80 grams [42]. This value is close to an averaged serving of fruits and vegetables. Considering that frequency of intake could be more important than the serving size, to classify individuals as having a minimum intake of 400 grams of fruits and vegetables per day, these potential biases are not likely to affect the results.

A systematic review of the effectiveness of the program of five-a-day identified an increase from 0.1 to 1.4 servings per day in a setting of primary prevention [43]. Adolescents from Southern Brazil already have that average consumption of fruits and vegetables, suggesting that the target program’s five-a-day can be more easily achieved. In addition to increasing consumption of fruits and vegetables, young people should be informed that rice and beans are not part of five servings per day. Finally, the diversity of fruits and vegetables can be an advantage to be explored in a five-a-day program.

In conclusion, adolescents from southern Brazil have a low frequency of five servings a day of combined fruits and vegetables. The detailed description of the current pattern of diet by adolescent may guide a strategy to recommend five-a-day in the country. The data about adolescents from developing countries helps to fulfill a gap on intake of fruits and vegetables. Our study provides information for adolescents from a representative population-based sampling from southern Brazil, and dietary intake was acquired through rigorous epidemiological methodology.

## Abbreviations

Five-a-day: Five servings of fruits and vegetables per day, with or without rice and beans; Five-a-day – the color way: Five different servings of fruits and vegetables per day, with or without rice and beans; IPAQ: International Physical Activity Questionnaire; BMI: Body mass index; FFQ: Food frequency questionnaire; SOFT study: Syndrome of Obesity and Risk Factors for Cardiovascular Disease.

## Competing interests

None of the author has competing interests to declare.

## Authors’ contributions

MAR, MBM, LBM, FDF and SCF took part in the design, statistical analysis, and writing the manuscript. SCF conceived and coordinated the SOFT study. All authors read and approved the final manuscript.
